# 4-Bromo-*N*-(4-meth­oxy-2-nitro­phen­yl)benzamide

**DOI:** 10.1107/S1600536812010963

**Published:** 2012-03-31

**Authors:** Weerawat Sripet, Suchada Chantrapromma, Pumsak Ruanwas, Hoong-Kun Fun

**Affiliations:** a Crystal Materials Research Unit, Department of Chemistry, Faculty of Science, Prince of Songkla University, Hat-Yai, Songkhla 90112, Thailand; b X-ray Crystallography Unit, School of Physics, Universiti Sains Malaysia, 11800 USM, Penang, Malaysia

## Abstract

In the title compound, C_14_H_11_BrN_2_O_4_, the amide segment makes dihedral angles of 23.4 (2) and 20.5 (2)° with the benzene rings, while the dihedral angle between the bezene rings is 2.90 (8)°. The nitro and meth­oxy groups are almost coplanar with their bound benzene ring, with the r.m.s. deviation for the 11 non-H atoms being 0.0265 (1) Å. An intra­molecular N—H⋯O hydrogen bond generates an *S*(6) ring motif. In the crystal, mol­ecules are linked into [2-10] chains by weak C—H⋯O and C—H⋯Br inter­actions, which form an *R*_2_^2^(8) motif between pairs of mol­ecules in the chain. A Br⋯O [3.2018 (12) Å] short contact also occurs.

## Related literature

For hydrogen-bond motifs, see: Bernstein *et al.* (1995 ▶). For related structures, see: Johnston & Taylor (2011 ▶); Li & Cui (2011 ▶); Saeed *et al.* (2008) ▶. For the stability of the temperature controller used in the data collection, see Cosier & Glazer (1986 ▶). For standard bond lengths, see: Allen *et al.* (1987 ▶).
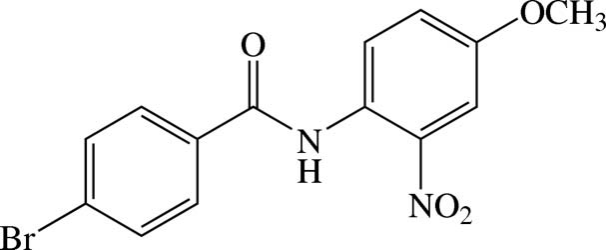


## Experimental

### 

#### Crystal data

C_14_H_11_BrN_2_O_4_
*M* *_r_* = 351.15
Triclinic, 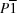

*a* = 6.1219 (2) Å
*b* = 7.6519 (3) Å
*c* = 14.3504 (6) Å
α = 89.197 (1)°
β = 84.795 (1)°
γ = 77.983 (1)°
*V* = 654.78 (4) Å^3^
*Z* = 2
Mo *K*α radiation
μ = 3.16 mm^−1^
*T* = 100 K
0.54 × 0.27 × 0.17 mm

#### Data collection

Bruker APEX DUO CCD area-detector diffractometer
Absorption correction: multi-scan (*SADABS*; Bruker, 2009 ▶) *T*_min_ = 0.281, *T*_max_ = 0.616
14195 measured reflections
3725 independent reflections
3558 reflections with *I* > 2σ(*I*)
*R*_int_ = 0.022

#### Refinement

*R*[*F* ^2^ > 2σ(*F* ^2^)] = 0.026
*wR*(*F* ^2^) = 0.080
*S* = 1.12
3725 reflections
195 parameters
H atoms treated by a mixture of independent and constrained refinement
Δρ_max_ = 0.93 e Å^−3^
Δρ_min_ = −0.48 e Å^−3^

### 

Data collection: *APEX2* (Bruker, 2009 ▶); cell refinement: *SAINT* (Bruker, 2009 ▶); data reduction: *SAINT*; program(s) used to solve structure: *SHELXTL* (Sheldrick, 2008 ▶); program(s) used to refine structure: *SHELXTL*; molecular graphics: *SHELXTL*; software used to prepare material for publication: *SHELXTL* and *PLATON* (Spek, 2009 ▶).

## Supplementary Material

Crystal structure: contains datablock(s) global, I. DOI: 10.1107/S1600536812010963/hb6654sup1.cif

Structure factors: contains datablock(s) I. DOI: 10.1107/S1600536812010963/hb6654Isup2.hkl

Supplementary material file. DOI: 10.1107/S1600536812010963/hb6654Isup3.cml

Additional supplementary materials:  crystallographic information; 3D view; checkCIF report

## Figures and Tables

**Table 1:** Hydrogen-bond geometry (Å, °)

*D*—H⋯*A*	*D*—H	H⋯*A*	*D*⋯*A*	*D*—H⋯*A*
N1—H1*N*1⋯O2	0.84 (3)	1.99 (3)	2.6318 (19)	132 (2)
C3—H3*A*⋯O4^i^	0.95	2.57	3.475 (2)	160
C12—H12*A*⋯O1^ii^	0.95	2.41	3.358 (2)	172
C10—H10*A*⋯Br1^iii^	0.95	2.93	3.863 (2)	167

Symmetry codes: (i) 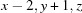; (ii) 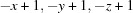; (iii) 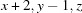.

**Fig. 1.**
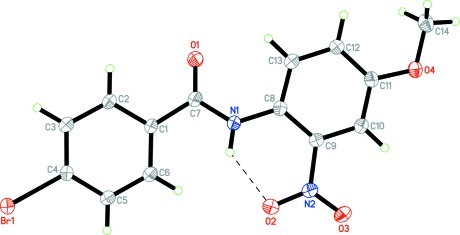
The molecular structure of the title compound, showing 30% probability displacement ellipsoids. The N—H···O hydrogen bond is drawn as dash line.

**Fig. 2.**
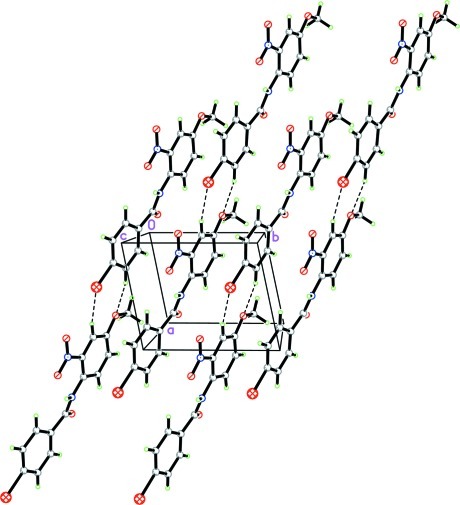
The crystal packing of the title compound viewed along the c axis. Hydrogen bonds were drawn as dashed lines.

## supplementary crystallographic information

### Comment

As part of our research in medicinal chemistry, the title benzamide derivative (I) was synthesized with a hope that it may exhibit anticancer and/or anti-alzheimer activities. Herein, its crystal structure was reported.

The molecule of the title benzamide derivative (Fig. 1), C_14_H_11_BrN_2_O_4_, is not planar as the plane of the middle N-C=O segment makes the dihedral angles of 23.4 (2) and 20.5 (2) ° with the C1–C6 and C8–C13 benzene rings, respectively whereas the dihedral angle between the two benzene rings is 2.90 (8)°. In the 4-methoxy-2-nitrophenyl moiety, the nitro and methoxy groups are co-planar with the bound benzene ring with the r.m.s. deviation of 0.0265 (1) Å for the eleven non-H atoms [C8–C14/N2/O2–O4] and the torsion angles O2–N2–C9–C8 = -3.8 (2)°, O3–N2–C9–C8 = 175.88 (15)° and C14–O4–C11–C12 = 3.5 (2)°. An intramolecular N1—H1N1···O2 hydrogen bond generates a S(6) ring motif (Bernstein *et al.*, 1995). Bond distances are comparable with those in related structures (Johnston & Taylor, 2011; Li & Cui, 2011 and Saeed *et al.*, 2008).

In the crystal (Fig. 2), the molecules are linked into [210] chains by weak C—H···O and C—H···Br interactions forming R_2_^2^(8) motifs. Br1···O2^iii^[3.2018 (12) Å; (iii) = -x, 2-y, -z] short contact is presented.

### Experimental

A mixture of 4-bromobenzoyl chloride (0.20 g, 0.91 mmol) and 4-metoxy-2-nitroaniline (0.23 g, 1.40 mmol) in anhydrous acetone (20 ml) was refluxed for 4 h. An orange solid was formed, which was filtered and washed with water. Orange blocks of the title compound were recrystallized from ethylacetate by slow evaporation of the solvent at room temperature after a week, Mp. 434-436 K.

### Refinement

Amide H atom was located in a difference map and refined isotropically. The remaining H atoms were positioned geometrically and allowed to ride on their parent atoms, with d(C-H) = 0.95 Å for aromatic and CH and 0.98 Å for CH_3_ atoms. The *U*_iso_ values were constrained to be 1.5*U*_eq_ of the carrier atom for methyl H atoms and 1.2*U*_eq_ for the remaining H atoms. A rotating group model was used for the methyl groups. The highest residual electron density peak is located at 0.75 Å from Br1 and the deepest hole is located at 0.85 Å from Br1.

### Crystal data

**Table:**

C_14_H_11_BrN_2_O_4_	*Z* = 2
*M**_r_* = 351.15	*F*(000) = 352
Triclinic, *P*1	*D*_x_ = 1.781 Mg m^−^^3^
Hall symbol: -P 1	Melting point = 434–436 K
*a* = 6.1219 (2) Å	Mo *K*α radiation, λ = 0.71073 Å
*b* = 7.6519 (3) Å	Cell parameters from 3725 reflections
*c* = 14.3504 (6) Å	θ = 2.9–30.0°
α = 89.197 (1)°	µ = 3.16 mm^−^^1^
β = 84.795 (1)°	*T* = 100 K
γ = 77.983 (1)°	Block, orange
*V* = 654.78 (4) Å^3^	0.54 × 0.27 × 0.17 mm

### Data collection

**Table:**

Bruker APEX DUO CCD area-detector diffractometer	3725 independent reflections
Radiation source: sealed tube	3558 reflections with *I* > 2σ(*I*)
Graphite monochromator	*R*_int_ = 0.022
φ and ω scans	θ_max_ = 30.0°, θ_min_ = 2.9°
Absorption correction: multi-scan (*SADABS*; Bruker, 2009)	*h* = −8→8
*T*_min_ = 0.281, *T*_max_ = 0.616	*k* = −10→10
14195 measured reflections	*l* = −20→20

### Refinement

**Table:**

Refinement on *F*^2^	Primary atom site location: structure-invariant direct methods
Least-squares matrix: full	Secondary atom site location: difference Fourier map
*R*[*F*^2^ > 2σ(*F*^2^)] = 0.026	Hydrogen site location: inferred from neighbouring sites
*wR*(*F*^2^) = 0.080	H atoms treated by a mixture of independent and constrained refinement
*S* = 1.12	*w* = 1/[σ^2^(*F*_o_^2^) + (0.0525*P*)^2^ + 0.2246*P*] where *P* = (*F*_o_^2^ + 2*F*_c_^2^)/3
3725 reflections	(Δ/σ)_max_ = 0.001
195 parameters	Δρ_max_ = 0.93 e Å^−^^3^
0 restraints	Δρ_min_ = −0.48 e Å^−^^3^

### Special details

**Table:**

Experimental. The crystal was placed in the cold stream of an Oxford Cryosystems Cobra open-flow nitrogen cryostat (Cosier & Glazer, 1986) operating at 100.0 (1) K.
Geometry. All esds (except the esd in the dihedral angle between two l.s. planes) are estimated using the full covariance matrix. The cell esds are taken into account individually in the estimation of esds in distances, angles and torsion angles; correlations between esds in cell parameters are only used when they are defined by crystal symmetry. An approximate (isotropic) treatment of cell esds is used for estimating esds involving l.s. planes.
Refinement. Refinement of F^2^ against ALL reflections. The weighted R-factor wR and goodness of fit S are based on F^2^, conventional R-factors R are based on F, with F set to zero for negative F^2^. The threshold expression of F^2^ > 2sigma(F^2^) is used only for calculating R-factors(gt) etc. and is not relevant to the choice of reflections for refinement. R-factors based on F^2^ are statistically about twice as large as those based on F, and R- factors based on ALL data will be even larger.

### Fractional atomic coordinates and isotropic or equivalent isotropic displacement parameters (Å^2^)

**Table:**

	*x*	*y*	*z*	*U*_iso_*/*U*_eq_	
Br1	−0.43080 (2)	1.27590 (2)	0.081247 (10)	0.02080 (7)	
O1	0.2430 (2)	0.77009 (18)	0.38411 (9)	0.0252 (3)	
O4	1.2328 (2)	0.25761 (17)	0.36805 (9)	0.0225 (2)	
O3	1.0773 (2)	0.5836 (2)	0.08860 (9)	0.0292 (3)	
O2	0.7257 (2)	0.70628 (18)	0.09318 (9)	0.0240 (3)	
N1	0.4593 (2)	0.69155 (19)	0.24641 (10)	0.0182 (3)	
N2	0.8886 (2)	0.61497 (18)	0.12882 (9)	0.0183 (3)	
C2	−0.1093 (3)	0.9574 (2)	0.28387 (11)	0.0183 (3)	
H2A	−0.1492	0.9142	0.3441	0.022*	
C3	−0.2720 (3)	1.0681 (2)	0.23633 (11)	0.0181 (3)	
H3A	−0.4229	1.0997	0.2629	0.022*	
C4	−0.2085 (3)	1.1314 (2)	0.14887 (11)	0.0165 (3)	
C5	0.0114 (3)	1.0909 (2)	0.10918 (11)	0.0178 (3)	
H5A	0.0518	1.1394	0.0504	0.021*	
C6	0.1719 (3)	0.9777 (2)	0.15721 (11)	0.0179 (3)	
H6A	0.3227	0.9473	0.1305	0.021*	
C1	0.1126 (3)	0.9086 (2)	0.24441 (11)	0.0163 (3)	
C7	0.2764 (3)	0.7849 (2)	0.29928 (11)	0.0176 (3)	
C8	0.6493 (3)	0.5785 (2)	0.27797 (11)	0.0164 (3)	
C9	0.8567 (3)	0.5414 (2)	0.22300 (10)	0.0166 (3)	
C10	1.0464 (3)	0.4333 (2)	0.25465 (11)	0.0175 (3)	
H10A	1.1836	0.4113	0.2159	0.021*	
C11	1.0359 (3)	0.3573 (2)	0.34300 (11)	0.0173 (3)	
C12	0.8314 (3)	0.3867 (2)	0.39815 (11)	0.0187 (3)	
H12A	0.8215	0.3326	0.4579	0.022*	
C13	0.6427 (3)	0.4951 (2)	0.36556 (11)	0.0184 (3)	
H13A	0.5048	0.5132	0.4038	0.022*	
C14	1.2322 (3)	0.1852 (2)	0.46014 (13)	0.0249 (3)	
H14A	1.3862	0.1315	0.4731	0.037*	
H14B	1.1412	0.0937	0.4650	0.037*	
H14C	1.1689	0.2807	0.5057	0.037*	
H1N1	0.467 (4)	0.710 (4)	0.1886 (19)	0.028 (6)*	

### Atomic displacement parameters (Å^2^)

**Table:**

	*U*^11^	*U*^22^	*U*^33^	*U*^12^	*U*^13^	*U*^23^
Br1	0.01483 (10)	0.02630 (10)	0.01952 (10)	−0.00065 (6)	−0.00143 (6)	0.00490 (6)
O1	0.0244 (6)	0.0295 (6)	0.0164 (5)	0.0048 (5)	0.0024 (4)	0.0015 (5)
O4	0.0157 (6)	0.0283 (6)	0.0209 (6)	0.0013 (4)	−0.0021 (4)	0.0057 (4)
O3	0.0174 (6)	0.0415 (7)	0.0236 (6)	0.0013 (5)	0.0065 (5)	0.0085 (5)
O2	0.0190 (6)	0.0306 (6)	0.0189 (5)	0.0019 (5)	0.0001 (4)	0.0063 (5)
N1	0.0159 (6)	0.0215 (6)	0.0146 (6)	0.0012 (5)	0.0004 (5)	0.0015 (5)
N2	0.0187 (7)	0.0196 (6)	0.0156 (6)	−0.0027 (5)	0.0005 (5)	0.0015 (5)
C2	0.0176 (7)	0.0208 (7)	0.0153 (6)	−0.0020 (5)	0.0009 (5)	0.0004 (5)
C3	0.0138 (7)	0.0211 (7)	0.0180 (7)	−0.0015 (5)	0.0016 (5)	0.0001 (5)
C4	0.0136 (7)	0.0180 (6)	0.0171 (7)	−0.0012 (5)	−0.0011 (5)	0.0004 (5)
C5	0.0156 (7)	0.0196 (7)	0.0168 (7)	−0.0017 (5)	0.0018 (5)	0.0012 (5)
C6	0.0146 (7)	0.0195 (6)	0.0180 (7)	−0.0015 (5)	0.0020 (5)	0.0007 (5)
C1	0.0147 (7)	0.0165 (6)	0.0163 (6)	−0.0010 (5)	−0.0001 (5)	−0.0001 (5)
C7	0.0157 (7)	0.0172 (6)	0.0184 (7)	−0.0008 (5)	0.0002 (5)	0.0001 (5)
C8	0.0142 (7)	0.0174 (6)	0.0166 (6)	−0.0009 (5)	−0.0012 (5)	0.0000 (5)
C9	0.0172 (7)	0.0177 (6)	0.0142 (6)	−0.0032 (5)	0.0007 (5)	0.0009 (5)
C10	0.0142 (7)	0.0190 (6)	0.0185 (7)	−0.0025 (5)	0.0010 (5)	−0.0001 (5)
C11	0.0140 (7)	0.0182 (6)	0.0192 (7)	−0.0016 (5)	−0.0027 (5)	0.0005 (5)
C12	0.0191 (7)	0.0196 (6)	0.0160 (7)	−0.0016 (5)	−0.0002 (5)	0.0024 (5)
C13	0.0152 (7)	0.0208 (7)	0.0174 (7)	−0.0012 (5)	0.0013 (5)	0.0012 (5)
C14	0.0237 (9)	0.0254 (8)	0.0233 (8)	0.0014 (6)	−0.0053 (6)	0.0037 (6)

### Geometric parameters (Å, º)

**Table:**

Br1—C4	1.8975 (16)	C5—H5A	0.9500
O1—C7	1.224 (2)	C6—C1	1.399 (2)
O4—C11	1.3615 (19)	C6—H6A	0.9500
O4—C14	1.426 (2)	C1—C7	1.499 (2)
O3—N2	1.2220 (19)	C8—C13	1.403 (2)
O2—N2	1.2395 (19)	C8—C9	1.410 (2)
N1—C7	1.367 (2)	C9—C10	1.387 (2)
N1—C8	1.404 (2)	C10—C11	1.389 (2)
N1—H1N1	0.84 (3)	C10—H10A	0.9500
N2—C9	1.4692 (19)	C11—C12	1.397 (2)
C2—C3	1.390 (2)	C12—C13	1.389 (2)
C2—C1	1.400 (2)	C12—H12A	0.9500
C2—H2A	0.9500	C13—H13A	0.9500
C3—C4	1.391 (2)	C14—H14A	0.9800
C3—H3A	0.9500	C14—H14B	0.9800
C4—C5	1.387 (2)	C14—H14C	0.9800
C5—C6	1.394 (2)		
			
C11—O4—C14	117.04 (13)	O1—C7—C1	121.42 (14)
C7—N1—C8	127.66 (14)	N1—C7—C1	114.35 (13)
C7—N1—H1N1	117.3 (19)	C13—C8—N1	121.83 (14)
C8—N1—H1N1	114.8 (19)	C13—C8—C9	116.34 (14)
O3—N2—O2	122.41 (14)	N1—C8—C9	121.82 (14)
O3—N2—C9	118.06 (14)	C10—C9—C8	122.13 (14)
O2—N2—C9	119.53 (13)	C10—C9—N2	115.12 (13)
C3—C2—C1	121.01 (14)	C8—C9—N2	122.75 (14)
C3—C2—H2A	119.5	C9—C10—C11	120.04 (14)
C1—C2—H2A	119.5	C9—C10—H10A	120.0
C2—C3—C4	118.32 (14)	C11—C10—H10A	120.0
C2—C3—H3A	120.8	O4—C11—C10	115.23 (14)
C4—C3—H3A	120.8	O4—C11—C12	125.42 (14)
C5—C4—C3	122.18 (15)	C10—C11—C12	119.35 (14)
C5—C4—Br1	119.02 (11)	C13—C12—C11	119.98 (14)
C3—C4—Br1	118.80 (12)	C13—C12—H12A	120.0
C4—C5—C6	118.73 (14)	C11—C12—H12A	120.0
C4—C5—H5A	120.6	C12—C13—C8	122.10 (14)
C6—C5—H5A	120.6	C12—C13—H13A	119.0
C5—C6—C1	120.52 (14)	C8—C13—H13A	119.0
C5—C6—H6A	119.7	O4—C14—H14A	109.5
C1—C6—H6A	119.7	O4—C14—H14B	109.5
C6—C1—C2	119.17 (14)	H14A—C14—H14B	109.5
C6—C1—C7	123.21 (14)	O4—C14—H14C	109.5
C2—C1—C7	117.61 (13)	H14A—C14—H14C	109.5
O1—C7—N1	124.23 (15)	H14B—C14—H14C	109.5
			
C1—C2—C3—C4	−0.9 (2)	N1—C8—C9—C10	178.41 (14)
C2—C3—C4—C5	−1.5 (2)	C13—C8—C9—N2	178.41 (14)
C2—C3—C4—Br1	177.86 (12)	N1—C8—C9—N2	−1.0 (2)
C3—C4—C5—C6	2.4 (2)	O3—N2—C9—C10	−3.6 (2)
Br1—C4—C5—C6	−176.97 (12)	O2—N2—C9—C10	176.71 (14)
C4—C5—C6—C1	−0.9 (2)	O3—N2—C9—C8	175.88 (15)
C5—C6—C1—C2	−1.3 (2)	O2—N2—C9—C8	−3.8 (2)
C5—C6—C1—C7	179.40 (14)	C8—C9—C10—C11	0.2 (2)
C3—C2—C1—C6	2.2 (2)	N2—C9—C10—C11	179.64 (14)
C3—C2—C1—C7	−178.44 (14)	C14—O4—C11—C10	176.68 (14)
C8—N1—C7—O1	−6.9 (3)	C14—O4—C11—C12	−3.5 (2)
C8—N1—C7—C1	173.95 (14)	C9—C10—C11—O4	−178.33 (14)
C6—C1—C7—O1	157.07 (16)	C9—C10—C11—C12	1.8 (2)
C2—C1—C7—O1	−22.2 (2)	O4—C11—C12—C13	178.40 (15)
C6—C1—C7—N1	−23.7 (2)	C10—C11—C12—C13	−1.8 (2)
C2—C1—C7—N1	156.97 (15)	C11—C12—C13—C8	−0.3 (2)
C7—N1—C8—C13	23.9 (2)	N1—C8—C13—C12	−178.36 (15)
C7—N1—C8—C9	−156.70 (16)	C9—C8—C13—C12	2.2 (2)
C13—C8—C9—C10	−2.2 (2)		

### Hydrogen-bond geometry (Å, º)

**Table:**

*D*—H···*A*	*D*—H	H···*A*	*D*···*A*	*D*—H···*A*
N1—H1*N*1···O2	0.84 (3)	1.99 (3)	2.6318 (19)	132 (2)
C3—H3*A*···O4^i^	0.95	2.57	3.475 (2)	160
C12—H12*A*···O1^ii^	0.95	2.41	3.358 (2)	172
C10—H10*A*···Br1^iii^	0.95	2.93	3.863 (2)	167

Symmetry codes: (i) *x*−2, *y*+1, *z*; (ii) −*x*+1, −*y*+1, −*z*+1; (iii) *x*+2, *y*−1, *z*.
